# Bleeding and Thrombotic Issues during Extracorporeal Membrane Oxygenation

**DOI:** 10.3390/jcm11185375

**Published:** 2022-09-13

**Authors:** Marco Ranucci, Mauro Cotza, Umberto Di Dedda

**Affiliations:** Department of Cardiovascular Anesthesia and Intensive Care, IRCCS Policlinico San Donato, San Donato Milanese, 20097 Milan, Italy

Extracorporeal Membrane Oxygenation (ECMO) is an advanced life support modality for patients with respiratory or cardiac failure refractory to standard therapy. When the failing organ is the lung, the usual configuration is a veno-venous (v-v) approach, with double cannulation at the level of the femoral and jugular veins or a single venous cannulation through the use of double lumen cannulas. When the heart is the failing organ, a veno-arterial (v-a) cannulation is required. Venous cannula is usually placed in the femoral vein and the arterial cannula in the femoral artery, but other configurations are possible, especially in small infants and in post-cardiotomy ECMO. Respiratory failure in small infants is treated as well with a v-a configuration (jugular vein and carotid artery).

The choice of v-a ECMO instead of ventricular assist devices (VAD) in adults is generally guided by the presence of a biventricular dysfunction and by the availability of VAD systems and local expertise.

In general, the indications for an ECMO support include adult respiratory distress syndrome (ARDS), neonatal respiratory distress syndrome, cardiogenic shock of different origins (resuscitation after cardiac arrest, myocarditis, post-cardiotomy, pulmonary embolism, and others), but ECMO can be used even for supporting heart function during specific, high-risk interventional procedures (in the cath-lab and electrophysiology).

Regardless of the indication and the configuration, a common factor is that ECMO requires systemic anticoagulation to prevent clot formation inside the circuit and/or the patient. This is usually achieved with low doses of unfractionated heparin (UFH) to reach and maintain an antiFXa activity in the range of 0.3–0.7 IU/mL, but direct thrombin inhibitors (bivalirudin, argatroban) are suitable alternatives.

Bleeding and thrombosis are common complications of ECMO. A recent study including 358 patients [1] reported 44.7% hemorrhagic complications (26.8% minor and 17.9% major) and 22.9% thrombotic complications (15.6% venous and 11.2% arterial). The same study addressed predictors of mortality in ECMO, and a multivariable analysis showed that hemorrhage has a hazard ratio of 1.74 (95% confidence interval 1.24–2.43, *p* = 0.001) for mortality.

Many registry studies have highlighted that thrombotic and hemorrhagic complications are the main determinants of bad outcomes in ECMO patients. The nature of the ECMO itself can tip the balance towards the ones or the others. In post-cardiotomy ECMO, for example, there is an initial higher risk for bleeding, due to the recent cardiac surgery, the prolonged cardiopulmonary bypass, the consumption of platelets, fibrinogen, and soluble coagulation factors, and the residual effects of UFH used during surgery. In other conditions, where the patient’s profile is “procoagulant”, the focus is on thrombotic issues.

COVID-19 patients have a well-known procoagulant pattern, and once requiring a v-v ECMO, this should be considered. There is no general agreement on the need for a more pronounced systemic anticoagulation in COVID-19 ECMO patients; however, evidence exists that these patients may be at higher risk for thrombotic complications.

Roedl and associates [2] investigated 113 patients receiving v-v ECMO to treat ARDS related to influenza-related disease (61 patients) or COVID-19 pneumonia (52 patients). The two populations differed at baseline, with COVID-19 patients showing a higher risk profile (older age, larger body mass index, higher rate of hypertension, chronic kidney disease, and diabetes) leading to a non-significantly higher mortality (54% vs. 38% at 28 days).

During the ECMO course, COVID-19 patients had thrombotic complications in 40% of the cases vs. 13% in influenza patients (*p* = 0.001) and bleeding occurred in 96% of the COVID-19 patients vs. 77% in the influenza cohort (*p* = 0.004). Of notice, four patients (8%) in COVID-19 cohort suffered from pulmonary embolism vs. only one (1.8%) in the influenza cohort.

There are specific indications for ECMO where the hemostatic issues acquire a particular importance. Massive acute pulmonary embolism with obstructive heart failure is one of these. Giraud and associates [3] retrospectively studied 36 patients receiving v-a ECMO for this indication. In a quote of patients (36%), ECMO was preceded by thrombolysis. This introduced a serious risk factor for hemorrhage and mortality. Non-survivors had a pre-ECMO thrombolysis in 77% of the cases vs. 26% in survivors (*p* = 0.005). The rate of hemorrhagic events was 92% in non-survivors vs. 26% in survivors (*p* < 0.001). The link between thrombolysis, hemorrhagic complications, and mortality was confirmed by a sensitivity analysis where patients receiving ECMO + thrombolysis had a hemorrhagic event in 100% of the cases vs. 5% in those receiving ECMO only (*p* < 0.001). These findings suggest that we could probably consider different patterns of anticoagulation once a patient received thrombolysis before ECMO.

Other cross-talks exist between ECMO complications and hemostatic issues. Acute kidney injury (AKI) is frequent in ECMO patients. In a series of 103 v-v ECMO, Pilarczyk and associates [4] investigated the outcome of patients with or without stage 2–3 AKI. They obviously found a worse outcome in AKI patients; of interest, the main complications in the AKI cohort were infectious and hemorrhagic. Patients with ECMO experienced ear, nose, and throat bleeding in 80% of cases vs. 50% in the no-AKI cohort (*p* = 0.008), and pulmonary bleeding occurred in 29% vs. 11% in no-AKI cohort (*p* = 0.033). This higher rate of bleeding is probably to be attributed to the use of renal replacement therapy in the most severe AKI cases, leading to additional exposure of the patient to foreign surfaces.

The evidence of the high rate of hemorrhagic and thromboembolic complications during ECMO, and their role in determining bad outcome, introduces the complex issue of systemic anticoagulation protocols and monitoring.

In an interesting retrospective study, Rajsic and associates [5] investigated 321 patients who underwent ECMO for different indications (respiratory failure: 24%; non-surgical cardiac failure: 52%; post-cardiotomy: 19%; trauma: 1%; and others: 4%). All the patients received a loading dose of 50–100 IU of UFH (if they were not already on cardiopulmonary bypass), and the subsequent anticoagulation was titrated according to a number of monitoring tools (activated clotting time, ACT; aPTT; anti FXa; and CT at INTEM). UFH was initiated at a dose of 5–20 IU/kg/h and subsequently adjusted to maintain an aPTT of 50–70 s and an antiFXa of 0.3–0.5 µg/mL. In presence of severe coagulopathy, the anticoagulation was stopped. The authors report 19% major hemorrhage events, 19% minor hemorrhage events, 36.4% of any hemorrhage within the first three ECMO support days, and 23% of thrombotic events. At follow-up, after 13 ECMO days, the freedom from bleeding events was <50%. Of interest, patients with bleeding events had lower values of C-reactive protein and procalcitonin, whereas coagulation parameters were the same of non-bleeding patients, with the only exception of a higher antithrombin value. However, it must be considered that they received more fresh frozen plasma, more platelet concentrates, more fibrinogen concentrate and prothrombin complex concentrate, and more supplementation with FXIII and von Willebrand factor. Therefore, it is reasonable that they maintained a coagulation profile similar to non-bleeding patients due to a larger correction of the hemostatic profile. In a multivariable analysis, the risk factors for bleeding were the SAPS score, a low value of C-reactive protein, and a longer aPTT. The authors conclude that hyperinflammation, through the promotion of thrombin formation, may exert a sort of “protective effect” against bleeding, hypothesizing that in presence of a low inflammatory profile the anticoagulation regimen could be reduced.

One of the problems when structuring and following an anticoagulation protocol for ECMO is that different target measures are available (ACT, aPTT, antiFXa, and reaction times at viscoelastic tests), and their results are not always univocal. Moussa and associates [6] explored the association between antiFXa and aPTT in a series of 265 adult patients supported with v-a ECMO. AntiFXa and aPTT were concordant in 51% of the paired samples, with 39% of sub-therapeutic aPTT values. Different covariates were involved in the association between antiFXa and aPTT: fibrinogen, prothrombin time, factor V, platelet count, bilirubin, and LDH. Of note, there was no association of daily maximum, minimum, and mean value of antiFXa and aPTT with bleeding events. The same happened for association with thrombotic events. These data suggest that the standard anticoagulation monitoring has little value for predicting and modulating the risk of bleeding and thrombotic events.

To this respect, an interesting study [7] addressed the time-related changes in coagulation factors in a series of v-v ECMO patients with ARDS. The authors found an immediate decrease in FII, FV, FVII, FVIII, FIX, FX, FXI, and FXII values. FXIII had subnormal values (36%) before the onset of ECMO. During ECMO, all the coagulation factors remained stable or recovered; conversely, FXIII continued to decline and then recovered only after ECMO discontinuation. This peculiar behavior suggests that the measure of FXIII may be useful within the ECMO anticoagulation monitoring.

Finally, novel strategies to assess platelet function during ECMO have been proposed [8] to overcome the limitations of the current platelet function tests. Although interesting, the proposed flow cytometry assessment is difficult to hypothesize as a routine measure during ECMO.

In conclusion, bleeding and thrombosis on ECMO remain the main complication, and the search for an anticoagulation monitoring adequate to limit these complications still appears elusive.

## References

[1] Treml B., Breitkopf R., Bukumirić Z., Bachler M., Boesch J., Rajsic S. (2022). ECMO Predictors of Mortality: A 10-Year Referral Centre Experience. J. Clin. Med..

[2] Roedl K., Kahn A., Jarczak D., Fischer M., Boenisch O., de Heer G., Burdelski C., Frings D., Sensen B., Nierhaus A. (2021). Clinical Characteristics, Complications and Outcomes of Patients with Severe Acute Respiratory Distress Syndrome Related to COVID-19 or Influenza Requiring Extracorporeal Membrane Oxygenation-A Retrospective Cohort Study. J. Clin. Med..

[3] Giraud R., Laurencet M., Assouline B., De Charrière A., Banfi C., Bendjelid K. (2021). Can VA-ECMO Be Used as an Adequate Treatment in Massive Pulmonary Embolism?. J. Clin. Med..

[4] Pilarczyk K., Huenges K., Bewig B., Balke L., Cremer J., Haneya A., Panholzer B. (2022). Acute Kidney Injury in Patients with Severe ARDS Requiring Extracorporeal Membrane Oxygenation: Incidence, Prognostic Impact and Risk Factors. J. Clin. Med..

[5] Rajsic S., Breitkopf R., Oezpeker U.C., Bukumirić Z., Dobesberger M., Treml B. (2022). The Role of Excessive Anticoagulation and Missing Hyperinflammation in ECMO-Associated Bleeding. J. Clin. Med..

[6] Moussa M.D., Soquet J., Lamer A., Labreuche J., Gantois G., Dupont A., Abou-Arab O., Rousse N., Liu V., Brandt C. (2021). Evaluation of Anti-Activated Factor X Activity and Activated Partial Thromboplastin Time Relations and Their Association with Bleeding and Thrombosis during Veno-Arterial ECMO Support: A Retrospective Study. J. Clin. Med..

[7] Moerer O., Huber-Petersen J.F., Schaeper J., Binder C., Wand S. (2021). Factor XIII Activity Might Already Be Impaired before Veno-Venous ECMO in ARDS Patients: A Prospective, Observational Single-Center Cohort Study. J. Clin. Med..

[8] Mansour A., Roussel M., Gaussem P., Nédelec-Gac F., Pontis A., Flécher E., Bachelot-Loza C., Gouin-Thibault I. (2020). Platelet Functions During Extracorporeal Membrane Oxygenation. Platelet-Leukocyte Aggregates Analyzed by Flow Cytometry as a Promising Tool to Monitor Platelet Activation. J. Clin. Med..

